# Silicon, endophytes and secondary metabolites as grass defenses against mammalian herbivores

**DOI:** 10.3389/fpls.2014.00478

**Published:** 2014-09-17

**Authors:** Otso Huitu, Kristian M. Forbes, Marjo Helander, Riitta Julkunen-Tiitto, Xavier Lambin, Kari Saikkonen, Peter Stuart, Sini Sulkama, Sue Hartley

**Affiliations:** ^1^Suonenjoki Research Unit, Finnish Forest Research InstituteSuonenjoki, Finland; ^2^Section of Ecology, University of TurkuTurku, Finland; ^3^Department of Biology, University of Eastern FinlandJoensuu, Finland; ^4^School of Biological Sciences, University of AberdeenAberdeen, UK; ^5^Plant Protection, Agrifood Research FinlandJokioinen, Finland; ^6^Department of Biology, University of YorkYork, UK

**Keywords:** defense, endophytes, grasses, grazing, phenolics, secondary metabolites, silicon, voles

## Abstract

Grasses have been considered to primarily employ tolerance in lieu of defense in mitigating damage caused by herbivory. Yet a number of mechanisms have been identified in grasses, which may deter feeding by grazers. These include enhanced silicon uptake, hosting of toxin-producing endophytic fungi and induction of secondary metabolites. While these mechanisms have been individually studied, their synergistic responses to grazing, as well as their effects on grazers, are poorly known. A field experiment was carried out in 5 × 5 m outdoor enclosures to quantify phytochemical changes of either endophyte-infected (E+) or endophyte-free (E-) meadow fescue (*Schedonorus pratensis*) in response to medium intensity (corresponding with densities of ca. 1200 voles/ha for 5 weeks during 3 months) or heavy intensity (ca. 1200 voles/ha for 8 weeks during 3 months) grazing by a mammalian herbivore, the field vole (*Microtus agrestis*). A laboratory experiment was then conducted to evaluate the effects of endophyte infection status and grazing history of the grass diet on vole performance. As predicted, grazing increased foliar silicon content, by up to 13%. Grazing also increased foliar levels of phosphorous and several phenolic compounds, most notably those of the flavonols isorhamnetin-diglycoside and rhamnetin derivative. Silicon concentrations were consistently circa 16% higher in E+ grasses than in E-grasses, at all levels of grazing. Similarly, concentrations of chlorogenic acid derivative were found to be consistently higher in E+ than in E- grasses. Female voles maintained on heavily grazed grasses suffered higher mortality rates in the laboratory than female voles fed ungrazed grass, regardless of endophyte infection status. Our results conclusively demonstrate that, in addition to tolerance, grasses employ multi-tiered, effective defenses against mammalian grazers.

## INTRODUCTION

Grasses are highly tolerant to grazing by means of their rapid regrowth capacity, basal meristems, underground storage organs and tillering capacity (Dyer et al., 1991; Karban and Baldwin, 2007). Indeed, grasses have been considered to primarily rely on these tolerance traits in lieu of physical or chemical defenses to mitigate damage caused by herbivory. However, a number of mechanisms have been identified in grasses that may deter feeding by grazers, and hence act as defenses (Vicari and Bazely, 1993). The most prominent of these is enhanced silicon (Si) uptake in response to damage (McNaughton and Tarrants, 1983; Massey et al., 2007a).

Following herbivore damage, Si is drawn from the soil and deposited systemically in the cell walls or lumina of new shoots as silica bodies or phytoliths (Ma and Yamaji, 2006; Currie and Perry, 2007). These forms of Si are extremely hard, and thereby increase the abrasiveness of leaf material (Massey and Hartley, 2006), potentially leading to accelerated tooth wear in grazers. High Si levels also inhibit the absorption of nitrogen from digested plant material by mammalian herbivores (Massey and Hartley, 2006).

Grazers have often been observed to avoid plant material with high Si content (Gali-Muhtasib et al., 1992; Cotterill et al., 2007; Reynolds et al., 2012). However, the effects of Si on mammalian herbivore feeding behavior appear in many cases to be moderated or even over-ridden by a range of other factors including species identity (of both grass and herbivore), genotype or growing environment and other aspects of forage quality (Shewmaker et al., 1989; Massey et al., 2009; Soininen et al., 2013a).

Many grass species harbor systemic, vertically transmitted endophytic fungi, which may also increase the resistance of the host to herbivory (Clay, 1988; Cheplick and Clay, 1988; Saikkonen et al., 1998, 2006). Endophytic fungi are often producers of mycotoxins, which either reduce the palatability of the grass or render it toxic to the herbivore (Clay, 1988; Powell and Petroski, 1993; Siegel and Bush, 1996; Saikkonen et al., 1998, 2006). Both the fungal load of grasses and the amount of toxins they produce may increase in response to herbivory, and hence act as induced defenses (Bazely et al., 1997).

While adverse effects of endophyte consumption are well documented for domestic livestock (reviewed by Clay, 1988), results from studies on wild mammalian grazers are scarce and ambiguous at best. Some studies report negative effects, such as decreases in population density (Coley et al., 1995), increased toxicity-induced mortality (Conover, 1998) and suppression of reproduction and growth (Jackson et al., 1996; Durham and Tannenbaum, 1998; Fortier et al., 2000; Conover, 2003), while others have found no effects at all (Barger and Tannenbaum, 1998; Saari et al., 2010). Thus, the effects of endophytic fungi on herbivores may be dose-dependent and may vary between grass, fungi and herbivore species.

Grasses are known to produce an array of secondary metabolites, for example hydroxamic acids (Niemeyer, 1988), condensed tannins (Bernays et al., 1989), cyanogenic glycosides (Jones, 1998) and alkaloids, albeit at levels much lower than dicotyledons (Coughenour, 1985; Vicari and Bazely, 1993). Secondary metabolites found in grasses have been shown to have adverse effects on the performance of rodents when consumed in artificial diets (Lindroth and Batzli, 1984). However, only rarely have these compounds had measurable negative impacts on free-ranging mammalian herbivores at the concentrations in which they naturally occur in grasses. Exceptions again involve livestock: cyanogenic glycosides poisoning cattle (Georgiadis and McNaughton, 1988) and indole alkaloids reducing the palatability of grass for sheep (Simons and Marten, 1971; Marten et al., 1973).

Most studies have failed to identify significant effects of grazing on levels of defensive secondary metabolites in grasses (Lindroth and Batzli, 1986; Klemola et al., 2000). Nonetheless, graminivorous herbivores are highly selective feeders both within and between plant species (Freeland, 1974; Hjältén et al., 1996). As this behavior is to a great extent governed also by the secondary chemistry of their food plants (Jung and Batzli, 1981; Bergeron and Joudoin, 1989; Massey et al., 2007b), variation in grass secondary metabolite concentrations holds the potential to influence grazing mammals and their population demography (Freeland and Janzen, 1974).

Although these three potential defensive mechanisms of grasses – Si induction, endophytes, and secondary metabolites – have been individually studied, their synergistic responses to grazing and interactive effects on herbivores are poorly known. The aim of this study was to simultaneously quantify how levels of Si, other nutrients and secondary metabolite content interact in grasses in response to mammalian grazing and endophyte infection status. Furthermore, the aim was to evaluate the synergistic effect of these potential defense mechanisms on the physiological performance of a graminivorous herbivore. Although all of the grass defenses focussed on in this study are mechanisms through which herbivore populations may potentially be affected, the evidence for impacts of mechanisms in natural populations is missing.

Meadow fescue (*Scherodonus pratensis* ex. *Lolium pratense* and *Festuca pratensis*), which is commonly infected by the endophytic fungus *Epichloë uncinata* (Saari et al., 2009) was used as a model grass species. As an indicator of secondary metabolite production, we analyzed levels of phenolics, a widespread group of important defensive compounds (Rhoades, 1979) known to have adverse effects on graminivorous herbivores (Lindroth and Batzli, 1984). A common European grassland rodent, the field vole *Microtus agrestis* served as our model grazing herbivore.

The following specific hypotheses were tested: (1) vole grazing elevates levels of both Si and phenolics in grasses, (2) the magnitude of response of Si and phenolics to grazing differs between endophyte-infected and non-infected grasses, and (3) consumption of heavily grazed grass, as compared to consumption of less heavily grazed grasses, has negative impacts on vole physiological condition, and more so if the grass is endophyte-infected.

## MATERIALS AND METHODS

### STUDY SPECIES

Meadow fescue is one of the most important native forage grasses in Nordic countries. It is also common outside of agronomic use in meadows, roadsides, and wastelands (Hämet-Ahti et al., 1998). Meadow fescue cultivars are often colonized by the systemic endophyte *E. uncinata*, which produces loline alkaloids, which appear to be non-toxic to large mammal herbivores (Clay and Schardl, 2002) but can be noxious to invertebrates and small vertebrates (Conover, 2003; Saikkonen et al., 2006; Huitu et al., 2008). A common meadow fescue cultivar “Kasper,” registered and commercialized for use in Nordic countries in 1989 (Saari et al., 2009) was used for purposes of this study.

The field vole is a common and widespread small rodent species in Europe, including Fennoscandia (Myllymäki, 1977). It exhibits high-amplitude 3–4 year population cycles which are synchronous across large geographic areas (Sundell et al., 2004; Korpela et al., 2013). Field voles preferentially inhabit grassland habitats (Hansson, 1971; Myllymäki, 1977). Their diet consists predominately of grasses and forbs (Hansson, 1971).

### EXPERIMENTAL DESIGN AND PROTOCOL

**FIGURE 1:**
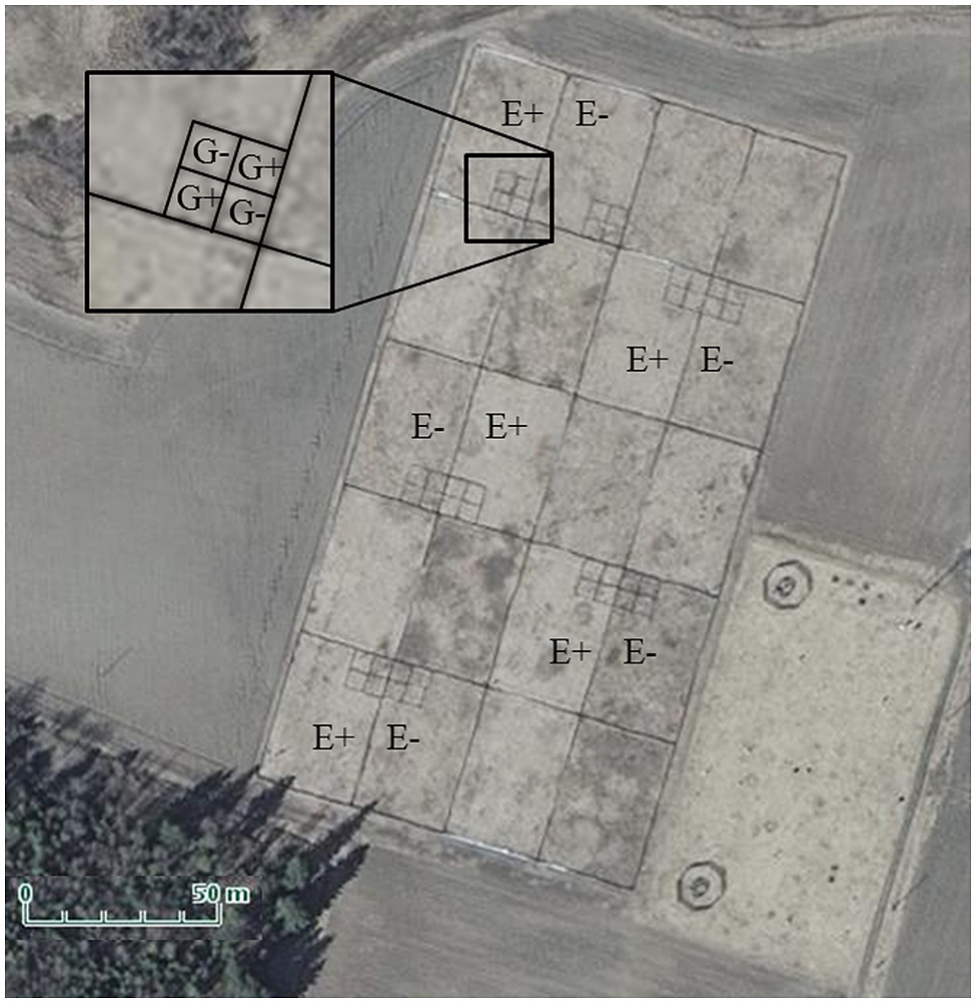
**Aerial photo of experimental enclosure complex.** The denotation E+ indicates those 25 × 39 m enclosures which were vegetated by endophyte-infected meadow fescue, and E- those with endophyte-free meadow fescue. The denotation G+ in the smaller plots pictured in the inset indicate those plots in which field voles grazed through the summer, and G- those, which were not grazed. Photo courtesy of the National Land Survey of Finland.

The study was conducted in fenced enclosures in Jokioinen, South–West Finland (60°49′ N, 23° 30′ E) during summer 2011. The enclosures (20 in total in 5 × 4 configuration, each 25 m × 39 m; **Figure 1**) were established in May 2006 in an old agricultural field by tilling, fertilizing with cow manure (30 000 kg/ha) and sowing as monocultures either endophyte-free seed lots (E-; 0% endophyte frequency) or endophyte-infected seed lots (E+; 79% infection frequency) of the meadow fescue cultivar “Kasper” at a rate of 20 kg ha^-1^ (Saikkonen et al., 2013). In June 2007, plots were fertilized again with a commercial fertilizer [16:9:22 (N:P:K) with micronutrients, Kemira, product number: 0647334]. E+ and E- treatments were randomly assigned to 10 enclosure pairs. Seed lots were obtained from seed production farms via the Finnish Food Safety Authority (EVIRA), Seed Certification Unit, Loimaa, Finland. Four years after establishment, the cover percentages of meadow fescue had decreased from 100 to 75% and 98% in E– and E+ plots, respectively, due to weed invasion (Saikkonen et al., 2013).

Each enclosure was constructed of a sheet metal fence (embedded 60 cm below ground while 60 cm remained above ground) in order to retain the experimental voles and exclude voles of natural populations and small mammal predators.

Every second enclosure pair of the 10 available pairs was selected for the experiment (**Figure 1**). Four 5m × 5 m sheet metal plots arranged in 2 × 2 squares were constructed (**Figure 1**) in one randomly assigned corner (corners bordering the outside of the enclosure complex were excluded to reduce predator attraction) of each of the 10 enclosures (5 × E+ and 5 × E-). Two plots per enclosure were randomly designated as vole grazing plots (G+) and two as non-grazed control plots (G-).

In total, the experiment consisted of 10 replicates of each of the following factorial treatments: vole grazing in endophyte-infected plots (G+E+), vole grazing in endophyte-free plots (G+E-), no grazing in endophyte-infected plots (G-E+), and no grazing in endophyte-free plots (G-E-). The grazing treatment was begun in late May 2011 by introducing two adult field voles, trapped in the vicinity of the enclosures and in Suonenjoki, Eastern Finland, into each G+ plot. Prior to introduction, all voles were housed in standard laboratory rodent cages (43cm × 26cm × 15 cm; Tecniplast, Italy) with ad libitum water, feed pellets (Altromin 1314F; Altromin Spezialfutter GmbH and Co. Germany), and turnip.

Two levels of grazing intensity we applied to ensure adequate responses of grasses to the grazing treatment. The medium grazing intensity treatment involved two voles grazing in each plot continuously for 2 weeks, and thereafter four voles grazing for 3 weeks, either directly after the first 2 weeks, or after a 3 week period of no grazing. The heavy grazing intensity treatment involved two voles grazing in each plot continuously for 2 weeks, and thereafter four voles grazing first for 3 weeks, followed by 3 weeks of no grazing and finally another 3 weeks of grazing by four voles.

In summary, the medium grazing intensity plots received 14 days of grazing at a vole density corresponding to 800 voles / ha (2 voles × 0.0025 ha = 800 voles/ha) plus 21 days of grazing at a vole density corresponding with 1600 voles/ha (4 voles × 0.0025 ha = 1600 voles/ha). The high intensity grazing treatments received 14 days of grazing at 800 voles / ha plus 42 days of grazing at 1600 voles/ha. The collective grazing intensity over the experiment approximated 1200 voles/ha for 5 weeks during 3 months and ca. 1200 voles / ha for 8 weeks during 3 months for the medium and heavy grazing intensity treatments, respectively. The level of grazing on individual grasses was not measured, but visual inspection indicated that while heavy grazing did have a substantial effect on vegetation, grasses were at no point during the experiment entirely deplete within the plots.

Live trapping was conducted in mid-June in all enclosures to measure the body mass of voles in the G+ enclosures and to verify that no voles had entered the G- enclosures. As expected, no voles were encountered in the G- enclosures. However, only circa 25% of the originally introduced voles were trapped in the G+ enclosures over three nights. This strongly indicated predation within the experimental enclosures, and a cat was occasionally seen around the enclosure complex.

Therefore, the experimental grazing protocol was altered from its original form. In mid-June, all voles were removed from the plots. Four field voles were then introduced into half of the G+ plots for a period of 3 weeks. Thereafter, the voles were trapped and translocated to the remaining plots. This was again repeated after 3 weeks, with four voles returned to the plots that were first grazed. During this 9 week period, only seven voles disappeared, and those that did were replaced upon translocation. All voles were live trapped out of the plots at the end of August.

### SAMPLE COLLECTION AND PHYTOCHEMICAL ANALYSES

To evaluate the effects of grazing on phytochemistry, meadow fescue samples were collected in each of the plots three times during the summer: immediately prior to introduction of voles, once at the beginning of August and once at the beginning of September (1 week after voles had been removed). Each time, one tiller was removed at the base from three randomly selected meadow fescue plants per plot and pooled into one sample. During the first sampling occasion, samples from the two G+ and two G- plots per enclosure were also pooled.

The Si, phosphorous (P), nitrogen (N), and carbon (C) contents of the samples were analyzed for each of the three sampling occasions. For these analyses, grass samples were washed under water, oven dried at 80°C and ground into a fine, non-fibrous powder. Three hundred milligram of this powder was pressed in a hydraulic press into a 3 mm thick, 13 mm diameter pellet with a pressure of 13 bar. Si and P content (expressed as percentage dry weight) was analyzed from the pellets with a portable X-ray fluorescence spectrometer (Niton XL3t900 GOLDD Analyzer; Thermo Scientific, Winchester, UK), as described by Reidinger et al. (2012). As this technique is non-destructible, the same pellets were used for the analysis of N and C. This was done by ISO 13878 and ISO 10694 based methods, respectively, using a LECO/CHN analyser (Leco Co., USA).

Phenolic compounds were analyzed only from samples collected on the last occasion, after the grazing treatment. These samples were air dried at room temperature for 1 week prior to analyses. All soluble non-tannin phenolic compounds were extracted and HPLC-analyzed from the grass leaf samples according to the protocol described in Nybakken et al. (2012).

### VOLE LABORATORY EXPERIMENT

At the beginning of September, a laboratory experiment was carried out to evaluate how earlier grazing and endophyte infection status affected vole physiological condition and performance. 96 wild field voles, captured near the towns of Suonenjoki, central Finland (62° N, 27° E) and Jokioinen, were housed in same sex pairs in a decommissioned greenhouse, in standard laboratory cages containing peat and straw as bedding and nest material. The voles were assigned to one of four food treatment groups (*n* = 24 voles per treatment group), which received *ad libitum* grass collected daily from the experimental plots (treatments: E+G+, E+G-, E-G+, and E-G-). For this segment of the study, all grass collected from grazed enclosures was pooled and not distinguished between heavy or medium grazing. Voles also received pieces of potato for extra hydration.

Experimental feeding began on September 10th and continued until the conclusion of the experiment on September 26th. Throughout this period, voles were checked daily for survival, and each animal was weighed using a spring balance (Pesola AG, Switzerland; accuracy ± 1 g) every 3–4 days (six measurement occasions in total). In the beginning and at the end of the feeding period, a blood sample was collected from the retro-orbital sinus of each vole using a capillary tube. Tubes were then centrifuged at 12000 g for five minutes, and haematocrit expressed as the percentage of packed red blood cells in total volume. The feeding experiment was terminated and all voles euthanized due to an increased rate of mortality in the end of the second experimental week, for reasons that remain unidentified.

The experiment was carried out under permit from the Animal Ethics Council of the State Provincial Office of Southern Finland.

### STATISTICAL ANALYSES

Variation in grass Si, P, N, and C content was analyzed with linear mixed models using the maximum likelihood method, where each of the elements was in turn the response variable. Grazing intensity (heavy, medium or control), endophyte infection status (E+ or E-), time (June, August, September) and all interactions were initially entered in the models as class explanatory variables. Time was designated a repeated factor, with plot as the subject. In all cases, AIC-based model selection (Burnham and Anderson, 2000) indicated unstructured as the most parsimonious covariance type. Enclosure was entered as a random factor.

Variation in phenolics concentrations at the end of summer was also analyzed with separate linear mixed models for each compound using the maximum likelihood method. Initially, all peak intensity values for the different compounds were log_e_ -transformed. Grazing intensity, endophyte infection status and their interaction were entered as fixed explanatory variables, while enclosure and plot (nested within enclosure) were designated random factors.

The overall response of meadow fescue to the treatments in terms of their phenolic content was also analyzed with multivariate analysis of variance using all 16 identified phenolic compounds as response variables, and grazing intensity, endophyte infection status and their interaction as explanatory variables.

Changes in the body mass and haematocrit value of voles that survived to the end of the laboratory experiment were analyzed with linear mixed models, with the percentage of change relative to the beginning of the experiment as the response variable. Grazing history (grazed or not) and endophyte infection status of the grass they were fed, vole sex and body mass at the beginning of the experiment, together with all possible three-way interactions, were entered as fixed explanatory variables. Identity of the cage in which the voles were housed was entered as a random factor. Full models were reduced by removing explanatory terms on the basis of AIC. Model fit was assessed by visual observation of the final model residuals.

Vole survival during the laboratory experiment was analyzed with generalized linear mixed models, using vole survival as a binary response variable with a logit link. Grazing history and endophyte infection status of the grass, vole sex, and all interactions, were entered as fixed explanatory variables and vole cage as a random factor.

Full models were reduced in all analyses by removing explanatory terms on the basis of AIC – terms were removed if this reduced the AIC value by >2 units (Burnham and Anderson, 2000). Model fit was assessed in the linear mixed models by visual inspection of the final model residuals, and by the ratio of the generalized chi-square statistic and its degrees of freedom in the generalized linear mixed model. All analyses were carried out with SAS statistical software v9.3 (SAS^®^ Institute Inc., Cary, NC, USA).

## RESULTS

**FIGURE 2:**
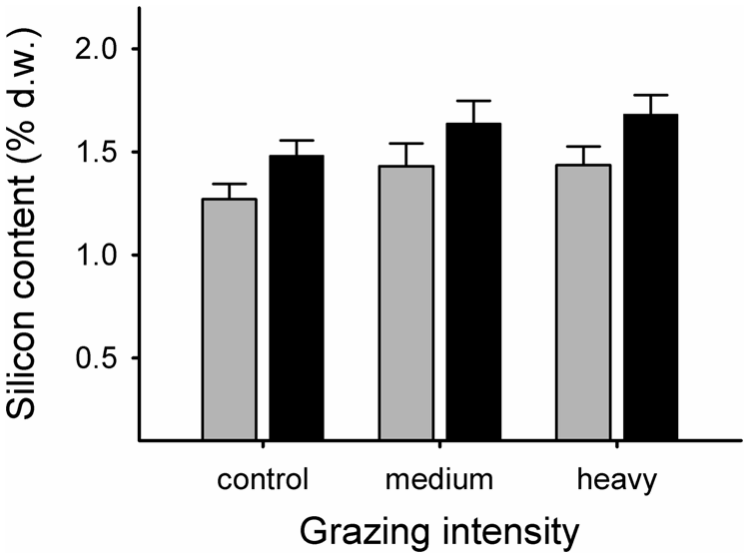
**Silicon content, expressed as percent dry weight in endophyte-free (E-; gray bars) and endophyte-infected (E+; black bars) grasses from control, medium grazing and heavy grazing intensity enclosures at end of summer.** Differences between grazing intensity treatments: *t*^control vs. heavy^_18.1_ = -2.38, *p* = 0.028; *t*^control vs. medium^_28.5_ = -1.83, *p* = 0.079; *t*^heavy vs. medium^_30.3_ = 0.24, *p* = 0.81. Differences in Si content between endophyte-infected and non-infected grasses: *F*^endophyte^_1,9.33_ = 6.97, *p* = 0.026).

Grazing elevated the Si content of grasses (*F*_2,24.4_ = 3.50, *p* = 0.046). Highest levels were recorded in the heavy grazing treatment (mean ± SE. Si content as percentage dry weight = 1.56 ± 0.07%), followed by medium grazing (1.54 ± 0.08%), while control grasses had the lowest values (1.36 ± 0.05%; **Figure 2**). Si levels were consistently higher in E+ grasses than in E- grasses, with no significant interactive effect of grazing (E+: mean ± SE. Si content as percentage dry weight = 1.60 ± 0.06%; E- = 1.38 ± 0.06%; **Figure 2**).

Likewise, grazing elevated the P content of grasses (*F*_2,28.4_ = 3.78, *p* = 0.035). Highest levels were recorded in the medium grazing treatment (mean ± SE. P content as percentage dry weight = 0.42 ± 0.01%), followed by heavy grazing (0.42 ± 0.01%), while control grasses had the lowest values (0.40 ± 0.01%; *t*^control^ ^vs.^ ^heavy^_24.8_ = -2.03, *p* = 0.054; *t*^control^ ^vs.^ ^medium^_31.2_ = -2.43, *p* = 0.021; *t*^heavy^ ^vs.^ ^medium^_30.5_ = -0.35, *p* = 0.73). The level of P declined in E+ grasses over the summer, whereas that in E- grasses remained stable (*F*_2,27.6_ = 3.79, *p* = 0.035). No differences were found in concentrations of N, C, or in C/N-ratio relative to grazing intensity, endophyte infection status, time or their interactions (all *p*-values > 0.08).

A two-way multivariate analysis of variance revealed a significant interaction between grazing intensity and endophyte infection status on the phenolics profile of the grasses (Wilks’ Lambda = 0.12, *F*_32,38_ = 2.27, *p* = 0.008). One-way multivariate tests with either E+ or E- samples did not clearly indicate which of the main effects had a stronger effect on grass phytochemistry. The varying degrees of grazing intensity tended to influence the phenolics profile of endophyte-free samples (Wilks’ Lambda = 0.001, *F*_32,4_ = 4.58, *p* = 0.074), but these differences were less pronounced in endophyte-infected samples (Wilks’ Lambda = 0.02, *F*_32,4_ = 2.40, *p* = 0.21).

Five of the 16 analyzed phenolic compounds exhibited an elevated response to grazing – chlorogenic acid, isorhamnetin-diglycoside, myricetin-glycoside, isorhamnetin 3-glucoside, and rhamnetin derivative (**Table 1**). Concentrations of chlorogenic acid derivative were higher in E+ than in E- grasses. Two compounds were affected by the interaction between grazing intensity and endophyte status. Levels of quercitrin, in particular, were positively associated with grazing intensity, but only in E+ grasses (**Table 1**).

**Table 1:** Mean peak intensity values of the phenolic compounds analyzed, grouped by grazing intensity, and endophyte status of the grasses.

	Grazing intensity							
Phenolic compound	Control		Medium		Heavy		Significant effect	Direction
	E-	E+	E-	E+	E-	E+		
Gentisic acid	0.12	0.10	0.08	0.09	0.09	0.10	n.s.	
Neochlorogenic acid	0.80	0.91	0.97	0.88	0.65	0.87	n.s.	
Chlorogenic acid	11.35	10.80	12.10	13.01	11.12	12.11	grazing	C < M (*p* = 0.057)
Chlorogenic acid derivative	0.12	0.17	0.13	0.16	0.11	0.15	endophyte	E- < E+, *p* = 0.051
Quercetin-diglycoside	1.36	1.12	1.37	1.42	1.23	1.34	n.s.	
Isorhamnetin-diglycoside	0.70	0.56	0.77	0.87	0.74	0.88	grazing	C < H and M
Myricetin-glycoside	0.01	0.01	0.01	0.02	0.03	0.04	grazing	H > C and M
Isorhamnetin-glycoside	0.02	0.03	0.03	0.04	0.06	0.05	n.s.	
Quercetin-glucoside	1.29	1.59	1.66	1.85	1.36	2.11	n.s.	
Kaempferol-glycoside	0.02	0.01	0.02	0.04	0.02	0.03	n.s.	
Quercitrin	0.03	0.01	0.03	0.05	0.03	0.06	interaction	C E- > C E+; H E+ > E-; C E+ < M E+ and H E+
Kaempferol 3-glucoside	0.07	0.10	0.11	0.11	0.09	0.11	interaction	C E- < M E-; C E- < M E+ (*p* = 0.054)
Isorhamnetin 3-glucoside	0.27	0.35	0.44	0.51	0.36	0.62	grazing	C < M and H
Rhamnetin derivative 1	0.20	0.20	0.21	0.23	0.20	0.23	n.s.	
Quercetin derivative	0.01	0.01	0.02	0.04	0.02	0.03	n.s.	
Rhamnetin derivative 2	0.11	0.12	0.13	0.15	0.12	0.14	grazing	C < M (*p* = 0.008) and H (*p* = 0.059)

*Significant effect denotes which of these terms was significant at a risk level of α = 0.05 (borderline values indicated in column Direction). Direction denotes pairwise differences between levels of the significant effect and their direction; C, M, and H denote control, medium and heavy grazing intensities, E+ and E- indicate endophyte-infected and non-infected grasses, respectively.*

**FIGURE 3:**
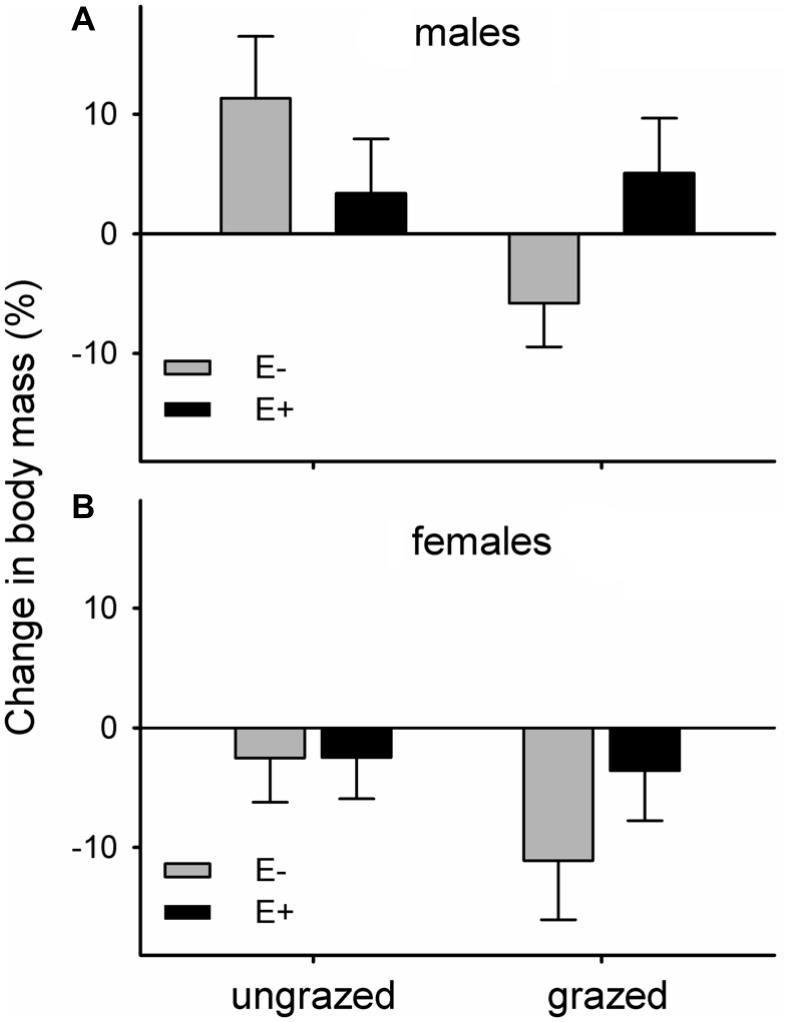
**Changes in body mass of voles maintained in the laboratory on previously grazed or ungrazed endophyte-infected (E+) or endophyte-free (E-) grass; (A) males, (B) females.** Differences of changes in body mass of voles relative to the treatments and vole sex: *F*^endophyte×grazing^_1,40.9_ = 4.14, *p* = 0.049; *F*^grazing^_1,38.8_ = 4.38, *p* = 0.043; *F*^sex^_1,40.3_ = 7.20, *p* = 0.01.

The body mass of voles maintained in the laboratory on endophyte-infected and endophyte-free grasses responded differently to the grazing history of their diet. Voles feeding on endophyte-free grass that had been grazed throughout the summer lost relatively more mass than voles in the other three groups (mean ± SE. mass change during experiment as percentage: E-G- = +3.90 ± 3.12%, E-G+ = -8.92 ± 3.08%, E+G- = +0.74 ± 2.88%, E+G+ = +0.62 ± 3.18%). Feeding on previously grazed grass also had a significant negative main effect on vole body mass change (mean ± SE. mass change: G- = + 2.32 ± 2.15%, G+ = -4.15 ± 2.18%). Females lost more body mass than males, irrespective of dietary group (mean ± SE. mass change: males = +3.21 ± 2.26, females = -5.04 ± 2.06%; **Figure 3**).

**FIGURE 4:**
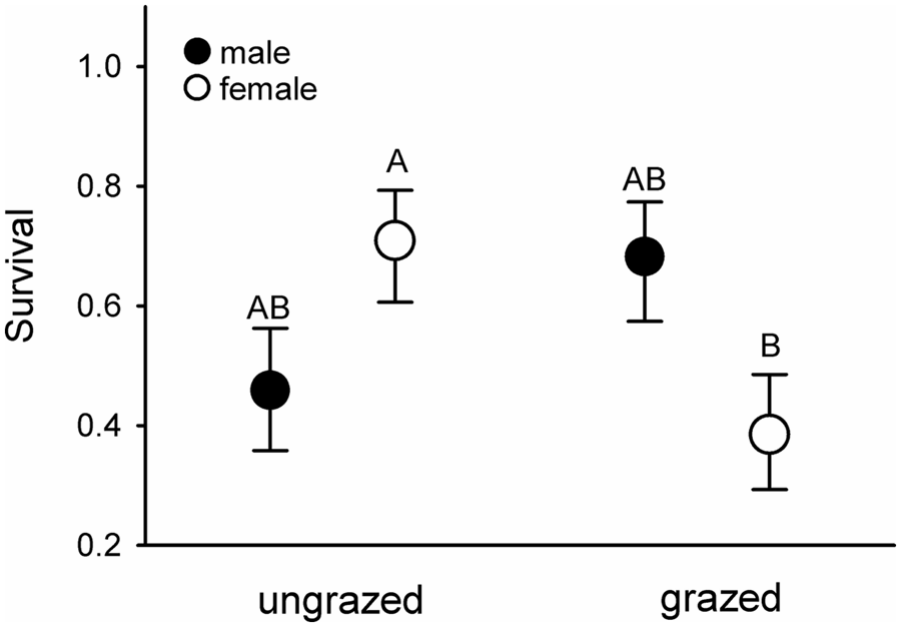
**Survival of male (filled symbols) and female (open symbols) voles maintained in the laboratory on previously ungrazed or grazed grass.** Letters above the symbols denote significant differences between the groups at α < 0.05. *F*^sex×grazing^_1,45.41_ = 6.83, *p* = 0.013.

Females that had fed on endophyte-infected grass had elevated haematocrit levels compared to those fed on endophyte-free grasses (mean ± SE.: E+ = 51.3 ± 0.8, E- = 48.9 ± 0.9; *F*_1,19.7_ = 4.55, *p* = 0.046). Conversely, females maintained on heavily grazed grasses suffered ca. 45% higher mortality rates than females fed ungrazed grass, regardless of endophyte infection status; these differences did not manifest in males (**Figure 4**).

## DISCUSSION

In support of our hypotheses, vole grazing elevated levels of both Si and phenolics in grasses. Endophyte infection alone also elevated grass Si content, to an extent comparable to that of grazing. However, changes in the levels of Si in response to grazing did not differ between E+ and E- grasses. Grazing and endophyte infection status did interact in altering the phenolic compound profile of grasses. Also in support of our hypothesis, consumption of heavily grazed grass had negative impacts on vole physiological condition, but these effects were conditional on endophyte infection status. Although endophyte infection elevates Si content as much as grazing alone does, our results overall suggest that grazing has a more prominent effect on grass defenses than endophyte infection status.

### GRASS RESPONSES TO GRAZING

As predicted, grazing-induced the uptake of Si from the soil into the grass. This response has been documented several times in association with various grazer taxa (McNaughton and Tarrants, 1983; Massey and Hartley, 2006; Massey et al., 2007a), and the biochemical processes underlying it are well known (Ma and Yamaji, 2006; Currie and Perry, 2007). Si accumulation has been identified as the most plausible, and potent defense mechanism of grasses against graminivorous voles (Massey and Hartley, 2006). It has even been suggested as a candidate factor for generating multiannual population cycles in rodents, as its induction in response to grazing may occur with a delay relative to rodent density, and the induced levels of Si persist in the grasses for sufficiently long (Massey et al., 2008; Reynolds et al., 2012).

Although the accumulation of Si has in many study systems received support as a defense mechanism against grazers, many others have failed to do so. For example, Shewmaker et al. (1989) could not demonstrate any effects of Si content on the grazing preference of sheep, which appear to be less responsive to variations in grass Si content than small rodents (Massey et al., 2009). Densities of African grazing ungulates have been shown to be either negatively (McNaughton and Tarrants, 1983) or positively (Georgiadis and McNaughton, 1990) associated with the Si content of their food plants. This contingency in responses is likely to reflect differences in grazing levels and hence in the magnitude of Si induction, given the known impacts of both frequency and intensity of damage on the Si response (Massey et al., 2007a; Reynolds et al., 2012), as well as variations in the range of plant, herbivore and environmental factors influencing plant-herbivore interactions (e.g., Soininen et al., 2013a).

In subarctic Fennoscandia, where vole densities are 2–3 orders of magnitude lower than simulated in this experiment, grass Si levels appear to be influenced more by grass species, their genotype or their growing environment than by the mostly low grazing pressure (Soininen et al., 2013a). Indeed, the majority of experimental studies in which Si has become induced in response to rodent grazing have been conducted in laboratory or greenhouse conditions (Massey and Hartley, 2006; Massey et al., 2007a; Reynolds et al., 2012, but see Massey et al., 2008), or as is the case here, in small outdoor enclosures. In these experimental conditions it is relatively easy to impose high damage levels in terms of biomass removed (Reynolds et al., 2012) or high vole densities (this study). It seems likely that both vole densities and the frequency and intensity of plant damage are likely to be lower in natural systems, which are also subject to a greater range of environmental variability (including climatic factors, soil type, plant genotypes, and growth stage), making the detection of Si induction in the field at the landscape scale more challenging.

While no differences were found in concentrations of N, C, or in C/N-ratio relative to grazing intensity or endophyte infection status, grazing did elevate the P content of grasses. Levels of phosphorous have been found to increase in response to grazing in grasses, but this primarily occurs to facilitate shoot regrowth after defoliation, rather than to act as an induced defense against grazers (Chapin and Slack, 1979; Donaldson et al., 1984; McNaughton and Chapin, 1985). It is evident that the primary chemistry of the grasses did not exhibit pronounced changes in response to defoliation by grazing.

Grazing increased the content of five of the 16 analyzed phenolic compounds – chlorogenic acid and five flavonols, isorhamnetin-diglycoside, myricetin-glycoside, isorhamnetin 3-glucoside, and rhamnetin derivative (**Table 1**). Many phenolics are inducible defensive compounds, which either inhibit digestion by herbivores, are deterrent or even directly toxic (Lindroth and Batzli, 1984; Karban and Baldwin, 2007). Their concentrations in grasses are generally low relative to other plant taxa (Bernays et al., 1989) and their collective levels appear not to respond greatly to grazing, at least by rodents (Lindroth and Batzli, 1986). However, in response to herbivory, the relative concentrations of different individual phenolic compounds may change dramatically without changing total concentrations, and it has been demonstrated that individual compounds can impact adversely on voles (Lindroth and Batzli, 1984).

A clear caveat of our study on grass phytochemical responses to grazing is the fact that concentrations of all possible defensive compounds, such as cyanogenic glycosides, hydroxamic acids, or alkaloids were not comprehensively analyzed. A number of these have been found to respond to grazing and to have adverse effects on herbivores (reviewed by Vicari and Bazely, 1993). The nature of these associations in our study system remains to be investigated in future research.

### EFFECTS OF ENDOPHYTE INFECTION ON GRASS PHYTOCHEMISTRY

Si levels were found to be to be slightly higher in E+ grasses than in E- grasses. Si has been shown to be positively associated with drought resistance in grasses, as it forms physical structures on leaves which reduce transpiration through stomata (Lux et al., 2002). Si is also known for its capacity to inhibit the growth of pathogenic fungi (Fauteux et al., 2005) and although often described as having mutualistic rather than antagonistic interactions with its host plants (Saikkonen et al., 2006), it is plausible that endophytic fungi are in some cases identified by their hosts as harmful pathogens (Saikkonen et al., 1998, 2004). An alternative explanation for the elevated Si levels observed in E+ grasses in this study may therefore be an evolutionarily conserved defensive response against the intrusion of a foreign fungal organism, even potentially mutualistic ones.

Similarly, chlorogenic acid derivative levels were higher in E+ than in E- grasses. Chlorogenic acid has been shown to act as a chemical defense against invertebrate herbivores (Bernays et al., 2000; Ikonen et al., 2001), though very little is known of how, or if, chlorogenic acid acts as a defense against fungal infections in plants, as Si does.

### INTERACTIVE EFFECTS OF GRAZING AND ENDOPHYTE INFECTION ON GRASS PHYTOCHEMISTRY

Two phenolic compounds were affected by the interaction between grazing intensity and endophyte status. Levels of quercitrin (also known as quercetin 3-rhamnoside), in particular, were positively associated with grazing intensity, but only in E+ grasses (**Table 1**). Quercitrin consumption has been shown to reduce the growth of weanling voles, and most profoundly so when dietary protein levels are low (Lindroth and Batzli, 1984). The effects of quercitrin on voles appear to be toxic, as dietary protein levels have been shown not to affect protein digestibility – rather, low dietary protein levels appear to reduce the capacity of voles to detoxify quercitrin (Lindroth and Batzli, 1984).

The grazing-induced elevation of Si may have more far-reaching effects than the up-regulation of the physical defense of plants. For example, Si appears also to have a functional role in the systemic response of plants to pathogens, through the activation of general defensive pathways, e.g., involving salicylic, or jasmonic acids (Fawe et al., 1998; Rodrigues et al., 2004; Fauteux et al., 2005). Elevated Si levels have also been shown to increase the accumulation of lignin, phenolic compounds, chitinases, and peroxidases (Fawe et al., 1998). As such, Si is likely to be integral in the regulation of terpene or phenolic –based defenses in stressed plants (Fauteux et al., 2005).

A caveat of our study is that we were unable to determine the effects of vole grazing on either the amount of endophyte hyphae per grass leaf or the concentrations of mycotoxin alkaloids that the fungus produces. Bazely et al. (1997) demonstrated that intensive sheep grazing increased hyphae loads and/or alkaloid production. A similar mechanism may occur with vole grazing, though this too remains a topic for further investigation.

### EFFECTS OF DIET QUALITY VARIATION ON VOLE CONDITION

Voles that consumed previously grazed grass lost body mass in the laboratory. Massey and Hartley (2006) established that an elevated concentration of Si in the diet is detrimental to graminivorous voles, in that it reduces their capacity to absorb nitrogen from their diet, leading to reduced growth rates in juveniles and adult females, and our study confirms this. Conversely, there are far fewer cases in which secondary metabolites have been shown to have similar negative effects on graminivorous mammals (see Simons and Marten, 1971; Marten et al., 1973; Georgiadis and McNaughton, 1988), let alone voles. Although grasses produce a wide array of secondary metabolites (reviewed by Vicari and Bazely, 1993), their effects on rodents appear to manifest only when animals are fed on experimental diets in laboratory conditions (e.g., Lindroth and Batzli, 1984). In light of this, it seems more parsimonious to attribute the observed body mass loss of voles to elevated Si content in the grasses than phenolics content. However, our experimental design does not enable us to firmly discriminate between these factors.

Females, in particular, suffered mass loss while maintained in the laboratory, regardless of treatment. The voles were offered only one species of grass as diet. Consumption of such a monotonous diet is highly unlikely for voles in natural habitats (Myllymäki, 1977; Soininen et al., 2013b). The fact that females suffered greater mass loss than males may be due to intersexual differences in dietary requirements related to, e.g., reproduction (Barboza and Bowyer, 2000; of note, none of the females in our experiment were gravid). Unfortunately, virtually no information exists on sex differences in dietary intake in rodents.

Females that had fed on endophyte-infected grass had elevated haematocrit levels compared to those fed on endophyte-free grasses. Haematocrit measures the relative volume of red blood cells in whole blood, and its values may increase due to either proliferation of red blood cells, or, more commonly, due to a reduction in blood plasma content due to dehydration (Fair et al., 2007). A dehydration-induced reduction in haematocrit often occurs in association with prolonged fasting in animals (Vleck et al., 2000). It is likely that the ultimate factors which lead to female mass loss and high death rate also contributed proximately to their haematocrit levels. Thereby, it is plausible that females were more sensitive either to the increased Si content of the E+ grasses, or to the endophyte itself, than were male voles.

Highest levels of mortality were recorded for females maintained on grazed grasses. Conversely, endophyte infection status did not affect mortality rates. Although endophyte infection status appeared to be associated with female food avoidance in the laboratory, as judged by hematocrit levels, these effects were not pronounced enough to impact survival. Collectively, our results indicate that grazing-induced changes in the quality of diet, i.e., increased Si and phenolics content, outweigh the potential negative effects of endophyte infection on the physiological condition of voles (see Lindroth and Batzli, 1984; Massey and Hartley, 2006). Furthermore, we demonstrate here that heavy and prolonged grazing may reduce the quality of graminivorous vole diet to the extent that it has negative effects on vole survival. By extension, as the most profound negative impacts were observed in females, these effects hold the potential to carry over to population growth. However, we acknowledge that the artificial nature of our laboratory may have generated spurious associations between food quality and vole performance. More detailed experimental research is therefore still called for to elucidate these causal relationships in entirely natural surroundings.

## CONCLUSION

Our results render support to the hypothesis that food quality may indeed have a limiting effect on vole population growth, in cases when grazing is severe and long enough (see Reynolds et al., 2012). We conclusively demonstrate here that grasses are capable of employing multi-tiered, effective defenses against mammalian grazers. We do not imply that these findings are universally applicable to all grazing ecosystems – rather, we highlight the need for more detailed investigations on the entire metabolome and the relative effects of defense mechanisms thus far identified in grasses to elucidate the role of food quality as a determinant of herbivore population dynamics.

## Conflict of Interest Statement

The authors declare that the research was conducted in the absence of any commercial or financial relationships that could be construed as a potential conflict of interest.
